# Detection of BRAF V600E Mutation in Ganglioglioma and Pilocytic Astrocytoma by Immunohistochemistry and Real-Time PCR-Based Idylla Test

**DOI:** 10.1155/2020/8880548

**Published:** 2020-08-17

**Authors:** Justyna Durślewicz, Anna Klimaszewska-Wiśniewska, Paulina Antosik, Anna Kasperska, Dariusz Grzanka, Tadeusz Szylberg, Łukasz Szylberg

**Affiliations:** ^1^Department of Clinical Pathomorphology, Nicolaus Copernicus University in Toruń, Faculty of Medicine, Collegium Medicum in Bydgoszcz, 85-092 Bydgoszcz, Poland; ^2^10th Military Research Hospital and Polyclinic, 85-681 Bydgoszcz, Poland

## Abstract

The BRAF V600E mutation is an important oncological target in certain central nervous system (CNS) tumors, for which a possible application of BRAF-targeted therapy grows continuously. In the present study, we aim to determine the prevalence of BRAF V600E mutations in a series of ganglioglioma (GG) and pilocytic astrocytoma (PA) cases. Simultaneously, we decided to verify whether the combination of fully automated tests—BRAF-VE1 immunohistochemistry (IHC) and Idylla BRAF mutation assay—may be useful to accurately predict it in the case of specified CNS tumors. The study included 49 formalin-fixed, paraffin-embedded tissues, of which 15 were GG and 34 PA. Immunohistochemistry with anti-BRAF V600E (VE1) antibody was performed on tissue sections using the VentanaBenchMark ULTRA platform. All positive or equivocal cases on IHC and selected negative ones were further assessed using the Idylla BRAF mutation assay coupled with the Idylla platform. The BRAF-VE1 IHC was positive in 6 (6/49; 12.3%) and negative in 39 samples (39/49; 79.6%). The interpretation of immunostaining results was complicated in 4 cases, of which 1 tested positive for the Idylla BRAF mutation assay. Therefore, the overall positivity rate was 14.3%. This included 2 cases of GG and 5 cases of PA. Our study found that BRAF V600E mutations are moderately frequent in PA and GG and that for these tumor entities, IHC VE1 is suitable for screening purposes, but all negative, equivocal, and weak positive cases should be further tested with molecular biology techniques, of which the Idylla system seems to be a promising tool.

## 1. Introduction

According to the National Cancer Institute, tumors of the central nervous system (CNS) represent only a small fraction of annual tumor incidence (1.4%) but are associated with a twice higher mortality rate (2.8%). In children under 14 years old, CNS tumors are the most frequent solid tumors, and half of the cases occur in infants 0-4 years old [1–3]. Brain tumors are a diverse group of neoplasms arising from different cells within the CNS or from primary tumors of other organs that spread to the CNS. Primary brain tumors include a number of histologic types with distinctly different tumor growth rates [4, 5].

Due to the uniqueness of the clinical material, we focused our attention on rare brain tumor types—ganglioglioma (GG) and pilocytic astrocytoma (PA). GGs are well-differentiated, rare CNS tumors, which are characterized by a slow, circumscribed growth and a relatively favorable prognosis. Gangliogliomas are generally benign WHO grade I tumors, most commonly located in the temporal lobes of children and young adults. However, they are both clinically and histologically heterogeneous, and tumor recurrence or anaplastic progression occurs in some cases [6]. PAs are a distinct histologic and biologic subset of gliomas that account for approximately 5.1% of all these tumors. It is the most common pediatric brain tumor in children [7]. Although preferentially located in the cerebellum, PA can arise anywhere in the CNS. Almost all are generally considered WHO grade I tumors [8].

One of the mutations that has aroused considerable interest in recent years concerns the *BRAF* (v-raf murine sarcoma viral oncogene homolog B) gene that encodes the protein belonging to a highly oncogenic RAS/RAF/MEK/ERK signaling pathway [9]. The serine/threonine protein kinase BRAF is an important player in the mitogen-activated protein kinase (MAPK) signaling pathway that transduces mitogenic signals from activated cell-surface growth factor receptors to the cell nucleus and as a result modulates many important cellular processes, such as tumor growth, differentiation, proliferation, and angiogenesis. Deregulation of these processes by oncogenic BRAF has been implicated in different mechanisms underlying cancer development and progression. Therefore, the BRAF pathway has become a molecular target for individualized cancer therapy, with promising results deriving from clinical trials [10].

The most frequent mutation is a single nucleotide substitution of thymine to adenine at nucleotide 1799 that converts valine (V) to glutamic acid (E) at amino acid 600 (V600E mutation) [11]. Other common types of *BRAF* mutations in codon 600 are *BRAF* V600E2, V600K, V600D, V600R, and V600M4 [12]. An alternative mechanism of MAPK activation is the formation of *BRAF* fusion genes. The most common fusion is between exon 16 of *KIAA1549* and exon 9 of *BRAF*, with less frequent fusion variants, including exon 16-exon 11 and exon 15-exon 9 [10, 13]. Currently, the *KIAA1549*-*BRAF* fusion accounts for around 58-75% of PA cases, so it is the most prevalent genetic alteration in this tumor entity. *BRAF* fusion results from tandem duplications or deletions on chromosome arms 7q.34 [3, 14–17].

The studies revealed that BRAF V600E mutation occurs in 100% of hairy cell leukemia [18], 50-60% of unresectable and metastatic malignant melanomas [19], approximately 30-50% of papillary thyroid carcinomas [20], 38% of Langerhans cell histiocytosis [21], 15-35% of serous low-grade and borderline ovarian carcinomas [22], 5-15% of colorectal adenocarcinomas [22], and 3-5% of non-small-cell lung carcinoma [9, 17–24]. Furthermore, BRAF mutations play an important role also in neurooncology [25]. The BRAF V600E mutation was observed in CNS tumors, e.g., in 66% of pleomorphic xanthoastrocytoma [26], 51% of dysembryoplastic neuroepithelial tumor [27], 18-57% of ganglioglioma [28], 5-15% of pilocytic astrocytoma [16, 17], and 1% of glioblastoma [26].

A crucial part of therapeutic strategies is rapid detection of a mutant protein. The immunohistochemical detection of the BRAF V600E mutation is possible using an antibody of choice. An alternative route is the use of existing molecular biology techniques to analyze point and other mutations in the *BRAF* gene. Among these techniques, the Idylla mutation tests coupled with the Idylla platform (Biocartis) have recently been proposed to serve as an attractive tool for the fast and easy detection of therapeutic markers, including *BRAF*. The Idylla BRAF mutation test is a fully automated, real-time PCR-based molecular diagnostics system, able to identify the presence of ≥1% *BRAF* V600E/E2/D/K/R/M-mutated cells in formalin-fixed, paraffin-embedded (FFPE) tumor tissue samples. Access to the sensitive and specific diagnostic tests and reliable tools to detect mutant proteins and genes is helpful in the implementation of appropriate therapeutic strategies.

Although in the case of certain CNS tumors the relevance of BRAF V600E mutation in the clinical setting has been increasingly acknowledged, a relatively low frequency of its occurrence requires further investigations and multiple experimental cohorts to establish its mutational status as a definitive biomarker for these tumors. Therefore, the primary aim of this study was to determine BRAF V600E mutation status in patients with rare CNS tumors—ganglioglioma and pilocytic astrocytoma. In addition, given that there is no standard method for testing *BRAF* status in diagnostic neurooncology [17], we aim to verify whether the combination of fully automated tests that are highly accessible for pathology laboratories—BRAF-VE1 IHC on VentanaBenchMark ULTRA platform and real-time PCR-based Idylla BRAF mutation assay—may be useful to accurately predict it in the FFPE tissues of GG and PA.

## 2. Material and Methods

### 2.1. Material

Archived FFPE tissues derived from 49 patients with tumors of the central nervous system, who were operated on in the 10th Military Research Hospital and Polyclinic, Bydgoszcz, Poland, between 2013 and 2018, were included in the present study. Histopathological diagnosis for each tumor sample was performed by two independent pathologists in the Department of Clinical Pathology, Collegium Medicum in Bydgoszcz, and in the Department of Pathology, 10th Military Research Hospital and Polyclinic. The study includes in total 15 gangliogliomas and 34 pilocytic astrocytomas. The median age at diagnosis was 28 (range: 9-76), and the overall male : female ratio was 25 : 24. There were 4 pediatric patients (<18 years; median age 15, range: 9-17), and others were adults (≥18 years, median age 28, range: 18-76). Representative tumor areas were selected in order to perform immunohistochemical and molecular tests on FFPE specimens.

### 2.2. Ethics Statement

The study protocol has been approved by The Ethics Committee of Nicolaus Copernicus University in Toruń, Ludwik Rydygier Collegium Medicum in Bydgoszcz (approval number KB 737/2019).

### 2.3. Methods

#### 2.3.1. Immunohistochemistry

Selected paraffin blocks were cut using a manual rotary microtome (Accu-Cut, Sakura, Torrance, CA, USA) to 3.0 *μ*m thick paraffin sections, placed on extra adhesive slides (SuperFrost Plus; Menzel-Glaser, Braunschweig, Germany), and dried at 60°C for 1 h. Subsequently, deparaffinization and rehydration were performed in EZ Prep solution (Ventana Medical Systems, Tucson, AZ, USA). Next, antigen retrieval of tissue sections was performed in a high pH Cell Conditioning (CC1) solution for 64 min. Immunohistochemical staining was done using the BenchMark®ULTRA automated slide processing system (Ventana Medical Systems, Tucson, AZ, USA) and visualized using the OptiView DAB IHC Detection Kit (Ventana Medical Systems, Tucson, AZ, USA), as recommended by the manufacturer. Incubation with the primary anti-BRAFV600E (VE1) antibody (Ventana Medical Systems, Tucson, AZ, USA) was performed for 40 min at 36°C. The slides were incubated with OptiView HQ Universal Linker and OptiView HRP Multimer and counterstained in hematoxylin and bluing reagent. Finally, the sections were dehydrated in increasing ethanol concentrations (80, 90, 96, and 99.8%), cleared in xylenes (I–IV), and sealed using a Dako mounting medium (Perlan, Inc., Santa Clara, CA, USA). Immunoreaction was labeled positive when unambiguous cytoplasmic staining of uniform or near-uniform intensity was seen and negative in the absence of staining or in the case of nuclear staining or when a staining of nontumor cells was observed. Of positive cases, the intensity of staining was scored as weak (1+), moderate (2+), or strong (3+). The criteria for equivocal immunoreaction included a faint staining indistinguishable from a nonspecific background staining, as well as highly heterogeneous cytoplasmic staining. The latter was defined as the presence of distinct subpopulations of tumor cells having a staining intensity that differed up to two scoring levels, e.g., one population of tumor cells with 3+ and another with 0 (negative). The scores for positive immunoreactivity were categorized as follows: 0: 0% of stained cell/area; 1 1-25% of stained cells/area; 2 26-50% of stained cells/area; 3 51-75% of stained cell/area; and 4 equal or more than 76% of stained cells/area.

#### 2.3.2. Idylla BRAF Mutation Test

Using the automated Idylla™ molecular diagnostics platform (Biocartis, Mechelen, Belgium), the selected specimens were tested for mutations in codon 600 of the *BRAF* gene. The Idylla™ platform is a real-time PCR and fluorophore-based detection system. Prior to the analysis, each sample was verified by independent pathologists, to confirm that at least 30% of tumor cells were present in every section. Selected paraffin blocks were cut using the manual rotary microtome (Accu-Cut; Sakura) to 10 *μ*m thick paraffin sections, which were placed between qualitative filter papers (10 mm in diameter), and then inserted to the Idylla™ BRAF mutation test cartridge (CE-IVD approved). For FFPE specimens, the sample preparation module uses high-intensity focused ultrasound technology to emulsify the paraffin and simultaneously rehydrate the tissue sample in an aqueous solution. Subsequently, isolated nucleic acids are transferred via microfluidic channels of the cartridge to appropriate separate PCR chambers with predeposited PCR reagents (i.e., primers, probes, and enzymes). To provide appropriate real-time PCR amplification and detection, all reagents are used in a dry form. Each PCR chamber allows for the identification of up to 6 different biomarker groups, each of which can be composed of multiple individual biomarkers. Once the sample is inserted into the cartridge and the lid is closed, the cartridge is hermetically sealed to eliminate risk of PCR contamination. The results, calculated by the dedicated Idylla™ software, were available after a 90 min run time. The test consists of three allele-specific PCR reactions that enable identification of *BRAF* wild-type, *BRAF* V600K/R/M (all c.1798G>A), or *BRAF* V600E/E2/D (all c.1799T>A) sequences (Table 1). The test enables identifying the presence of ≥1% *BRAF* V600 mutation in a background of wild-type allele. A quantification cycle (Cq) value is calculated by Idylla™ software for every valid PCR curve. The presence of a mutant genotype was determined based on the difference between Cq for wild-type *BRAF* and the V600E/E2/D or V600K/R/M Cq values. *BRAF* total, i.e., wild-type gene, was used as a sample processing control, and the melanoma specimen with established *BRAF* mutation served as a positive control. The mutant signal is considered valid if the *Δ*Cq is within a validated range. *BRAF* V600 mutation-negative samples were those for which a valid wild-type signal was observed but a *Δ*Cq value was outside the validated range [29, 30]. The interpretation of results is fully automatic with 4 possible results on the screen of the Idylla console: (i) no mutation detected in *BRAF* codon 600, (ii) mutation detected in *BRAF* codon 600, (iii) insufficient DNA input, and (iv) invalid.

## 3. Results

**Figure 1:**
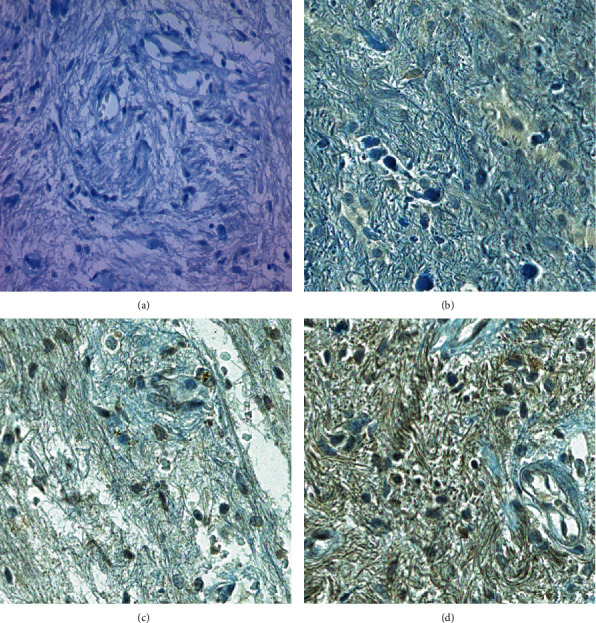
Immunohistochemical staining pattern of BRAF V600E (VE1) protein and negative control of the same case. Highly heterogeneous staining pattern is seen for PA case (no. 10), with different binding intensity—from clearly VE1-negative (b) tumor cells, through weak (c), to even strong (d). The image (a) of the same case stained with negative control is also seen. Magnification: 400x.

**Figure 2:**
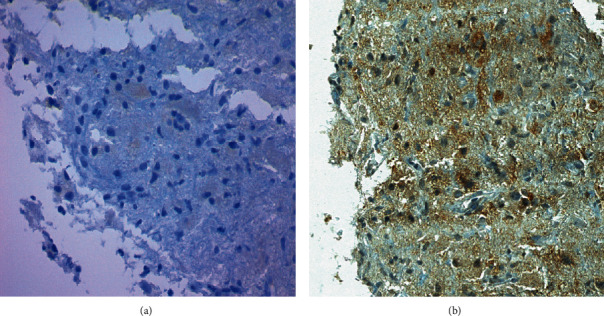
Negative control and immunohistochemical staining pattern of BRAF V600E (VE1) protein of the same case. Negative control (a) and nonspecific nuclear staining along with cytoplasmic staining in tumor cells (b) are seen. Magnification: 400x.

All 49 FFPE samples were examined for the presence of BRAF V600E mutation by IHC using the anti-BRAF V600E (VE1) antibody with the OptiView DAB IHC detection kit and the automated VentanaBenchMark® ULTRA platform. Of these samples, 6 (6/49; 12.3%) were scored as BRAF-VE1-positive, 4 (4/49; 8.2%) as equivocal, and 39 (39/49; 79.6%) as negative. Staining intensity was strong in 2 (2/6; 33,3%) cases, moderate in 3 (3/6; 50%) cases, and weak in 1 (1/6; 16.7%) sample. Half of the equivocal cases were GGs, and the other half were PAs. Both ambiguous cases of GG (nos. 2 and 3) were characterized by a small tumor cell content (30 and 10%) and cytoplasmic staining that was difficult to distinguish from nonspecific background staining. A similar situation, with respect to staining intensity, but not tumor cellularity (95%) was observed in one PA sample (no. 9). The other PA case (no. 10) was particularly complicated to interpret, due to a highly heterogeneous staining pattern. In these sections, there were the nests of VE1-positive tumor cells, with almost every subpopulation showing a different binding intensity—from weak to even strong, in addition to clearly VE1-negative (staining intensity 0) tumor cells (Figure 1). In addition, nonspecific nuclear staining along with cytoplasmic staining could be occasionally seen in tumor cells (Figure 2).

All cases positive or equivocal for BRAF V600E testing on immunohistochemistry were further assessed on the Idylla™ platform using the Idylla™ BRAF mutation assay. Of 6 positive BRAF-VE1 cases, all tested positive for *BRAF* V600E/E2/D mutation and none were positive for *BRAF* V600K/R/M mutation. Among these positive specimens, there was 1 (1/15; 6.7%) case of ganglioglioma and 5 (5/34; 14.7%) cases of pilocytic astrocytoma. Of 4 equivocal BRAF-VE1 samples, only 1 (no. 2) tested positive for *BRAF* V600E/E2/D mutation on the Idylla real-time PCR *BRAF* mutation test, whereas others were negative (each sample repeated twice). In addition, 12 (12/39; 30.8%) randomly selected cases from VE1-negative tumor samples, were further subjected to the Idylla™ *BRAF* mutation test. The molecular analysis confirmed their negative mutation status, with respect to not only *BRAF* V600E/E2/D mutations but also *BRAF* V600K/R/M mutations. Therefore, the overall positivity rate found in our study was 14.3% (7/49). This included 2 (2/15; 13.3%) cases of ganglioglioma and 5 (5/34; 14.7%) cases of pilocytic astrocytoma. The IHC-positive or equivocal cases ran on the Idylla real-time PCR instrument for the confirmation, and further assessment of *BRAF* V600 status is presented in Table 2.

F: female; M: male; IHC: immunohistochemistry; V600E/V600E2/V600D: presence of BRAF-V600E mutation detected by Idylla™; WT: wild type; IRS: the immunoreactive score of Remmele and Stegner, category of stained cells: 0: 0% of stained cell/areas; 1: 1-25% of stained cells/area; 2: 26-50% of stained cells/area; 3: 51-75% of stained cell/area; 4: equal or more than 76% of stained cells/area.

## 4. Discussion

The *BRAF* V600E mutation, among other molecular aberrations of this gene (in particular the presence of *KIAA1549-BRAF* fusions), is found in several CNS tumors, including gliomas and glioneuronal tumors. Although prognostic significance of *BRAF* alterations for these tumor entities is still inconclusive, a diagnostic utility for the differential diagnosis of pediatric gliomas, as well as a possible therapeutic utility, makes the assessment of their occurrence of growing clinical relevance [16]. Indeed, more and more case reports and ongoing clinical trials have been showing that CNS cancer patients harboring *BRAF* V600 mutations are responsive to BRAF (and MEK) inhibitors [31–33]. Therefore, it seems now clear that the diagnosis of CNS tumors should include molecular testing for *BRAF* mutations or fusions, and, if present, patients ought to be considered for targeted treatment [34].

The frequency of BRAF V600E mutations in GG and PA varies across scientific reports from 18 to 57% and from 5 to 15%, respectively [16, 17, 28]. However, in this topic, there is a relatively limited number of studies dedicated to these rare tumor types, compared with the multitude of research on, e.g., melanoma or papillary thyroid carcinoma. Hence, it was reasonable to assess BRAF V600E prevalence in an additional cohort. In relation to the largest study by Schindler et al. [35], who analyzed 1320 CNS tumor cases of pediatric and adult patients, including 77 GGs and 97 PAs of which 18% and 9% carried BRAF V600E mutation, we demonstrated a quite similar incidence rate, namely, 13.3% and 14.7%, respectively. This small but noticeable discrepancy may be due to the size of the research group, given that the small number of patients is undoubtedly a limitation of our study. However, a marked discordance exists between our results and those reported by Chappé et al. [36], Dougherty et al. [37], and Dahiya et al. [32], who observed the presence of BRAF V600E mutation in 45.2% (14/31), 50% (9/18), and 38.3% (18/47) of pediatric gangliogliomas, respectively. A high frequency of BRAF V600E mutations (58%) has been also revealed by Koelsche et al. [38] in a series of 71 pediatric and adult cases of GG (median age 22 years, range: 5-69). Importantly, they have demonstrated that BRAF V600E mutation is strongly associated with younger patient age and shows the highest frequency in the first decade of life, gradually decreasing thereafter. More specifically, GG patients with BRAF V600E mutations had a median age of 19 (range: 5-52) years at surgery, whereas patients with *BRAF* wild-type status were significantly older with a median of 31 years (range: 12-69). A similar age-dependent incidence of BRAF V600E mutations in GGs has been observed by Gierke et al. [16]. Furthermore, Myung et al. [39] have also demonstrated that the mutation was more common in pediatric GGs (34.7%) than adult counterparts (14.3%), but this relationship did not reach a statistical significance [39]. Our cohort included mostly adult patients (91.8% of cases, median age 28 years, range: 18-76); hence, discrepancies as to the frequency of BRAF mutations between the cited findings [17, 32, 36] and these presented here may be also related to this fact, apart from an obvious reason related to a low number (*n* = 15) of GG cases. In turn, a different pattern [16] or no association between patient age and BRAF V600E mutation [35, 40] has been found for pilocytic astrocytoma. In this context, Gierke et al. have shown that V600E mutations in PA were very scarce in the pediatric age group (2%; 1/45) and were limited to the middle age group (13%; 3/23) [16]. This could at least partially explain a higher rate of positive PA cases in our cohort as compared to the positivity rates previously reported in most of other studies (usually below 10%) [35, 40]. Our result (14.7%; 5/34) is close to the mutation rate (15.6%; 7/45) reported by Myung et al. [39] who, in addition, have shown that the presence of this mutation was not significantly related to patient age but was slightly more frequent in adult patients (17.6%) than pediatric ones (14.3%). Apart from different sample sets (varied in terms of, e.g., group size and age of patients), it should also be noted that the variations in the frequency of BRAF mutations between different studies, to the same extent, may also be attributed to methodology and equipment used.

The gold standard for BRAF mutation analysis is molecular biology (DNA-based) techniques, with classical Sanger sequencing being the most commonly used [41, 42]. However, in the case of CNS tumors, in particular ganglioglioma, the relevance of the latter method has recently been questioned [40, 43], due to its high detection threshold of 20% allele frequency, which does not work well with a low number and/or scattered distribution of neoplastic cells [44]. Although other molecular biology techniques, such as pyrosequencing, next-generation sequencing, PNA-clamping PCR, ddPCR, and ASqPCR, have higher sensitivity (detection of 0.02-10% mutant in a background of wild type), all DNA-based methods are often expensive, labor-intensive, time-consuming, and not widely available in pathology laboratories, also because they require a highly skilled operator and complex infrastructure [45, 46]. Immunohistochemistry with a recently developed mouse monoclonal mutation-specific antibody (VE1 cone) has shown promise as a more widely available, relatively inexpensive, and fast method, which allows a direct visualization of BRAF V600E protein at the single-cell level [47, 48]. Numerous studies on melanoma and papillary thyroid carcinoma have demonstrated an excellent concordance between VE1 IHC and molecular analysis (a sensitivity and specificity over 95%) [49–52]. However, the sensitivity and specificity of VE1 IHC in colorectal cancer range from 59 to 100% and 51 to 100%, respectively, and therefore, some of the studies concluded that this method should not be used to guide patient management in this disease entity [53]. In the case of CNS tumors, VE1 IHC was evaluated in several studies, in which it has been presented as an either excellent or suboptimal [54] technique in characterizing brain tumor tissue [25, 26, 40, 55].

Although sometimes burdened with some technical difficulties, the VE1 antibody has been suggested to be even superior to the sole use of sequencing in these tumor entities (especially GG). The reason is that VE1 immunostaining can be performed as part of a clinical routine, and it has been shown to detect BRAF V600E mutant GGs in sequencing-negative cases [40, 43]. Based on our results and those reported by other groups [54, 56], it seems that strong-to-moderate staining for the VE1 antibody in CNS tumors equates with the positive results of *BRAF* V600E molecular testing. Although in our study, this was also true for weak (1+) positive VE1 case, we feel that in daily practice such cases should be also labeled as those that require confirmation with molecular methods. It is mostly due to a nonspecific background staining that could be seen by us and others [54] with VE1 antibody in brain tumor tissues, making the interpretation of staining acquired on the sample ambiguous. In turn, no additional confirmation for BRAF V600E mutation appears necessary with completely negative VE1 results, as our randomly selected IHC-negative cases were also negative by molecular analysis. However, a lack of immunostaining does not rule out other mutation types at hotspot codon 600, which yet have a potential clinical implications, as patients with mutations other than V600E variant are also eligible to BRAF*-*targeted therapies [57]. In our study, we found 3 equivocal samples due to low staining intensity. Another sample was labeled “equivocal” based on strongly heterogeneous staining, which has also been previously described in the case of VE1 antibody in colon tumors, as the staining pattern recommended for proceeding to mutational assessment by molecular methods [48]. Only 1 of these IHC-equivocal cases tested positive for *BRAF* V600E mutation (no. 2). This was ganglioglioma, in which it was not possible to distinguish a weak immunostaining from a background staining. Thus, our study clearly shows that there is sometimes a thin line between a weak genuine VE1 immunostaining and nonspecific background staining, and taking into account the subjectivity of the examiners and their varied experience level, as well as the fact that this antibody recognizes only V600E variant, we suggest that the weak positive, equivocal, and negative cases on IHC should be further tested with molecular biology techniques. Simultaneously, this seems to be a good approach in scientific research, given that there are studies assuming a weak positive VE1 staining (1+) as negative [41, 58]. Collectively, our results highlight the importance of pursuing the occasional weak and equivocal results to further classification of mutational status. Therefore, we join the recent opinion of some authors that the utility of VE1 IHC in routine neuropathology should be limited to screening purposes [26]. This is also concordant with the recent recommendations for colorectal cancer and melanoma [41].

Given that BRAF-VE1 IHC in CNS tumor samples should be used in conjunction with molecular analysis, and that, as mentioned above, a conventional direct sequencing has a limited power in some of these samples, we decided to verify whether the Idylla *BRAF* mutation assay performed on the fully automated Idylla platform may be a useful tool to accurately predict *BRAF* mutation status in GG and PA. It seemed particularly justifiable in the case of these peculiar tumor tissues since the Idylla test has a benefit of high sensitivity with 1% detection limit, and simultaneously, it can be easily performed within approximately 90 min (with a hands-on time of less than 2 min), as part of a clinical routine in all pathology units without the necessity of additional staff with molecular biology expertise or extramolecular infrastructure. This is due to the fact that the Idylla technology is based on disposable cartridges that integrate all preprocessing steps (deparaffinization, tissue digestion, and DNA extraction), PCR thermocycling, and fluorescence detection in a single diagnostic test with all reagents on-board and an automated setup workflow. In addition, no posttest analysis is required, since the Idylla console autoanalyzed the PCR curves to qualitatively determine the *BRAF* mutation status [59, 60].

The Idylla BRAF mutation assay performed on the Idylla molecular diagnostics platform is an allele-specific real-time PCR-based technique, which has got CE-IVD certification for the qualitative detection of the V600E/E2/D and V600K/R/M mutations in FFPE samples of malignant melanoma [61–64]. It was also validated in thyroid tumor tissues [65] and colorectal cancer tissues [66]; however, to our knowledge, it has previously been tested only in few samples of CNS tissue [61, 67] but not in the case of GG tissues. In our study, all cases with positive results for BRAF V600E protein expression on IHC were tested positively using the Idylla™, which confirms the effectiveness of this system. Importantly (as mentioned above), we could also detect a *BRAF* V600E mutation in our IHC-equivocal FFPE sample (no. 2) that contained 30% tumor cells and, as such, did not meet the minimum tissue requirement for the Idylla *BRAF* mutation testing [60]. In the other 3 IHC-equivocal cases, the Idylla showed no mutation detected in *BRAF* codon 600. Although none of these samples produced invalid or insufficient DNA input calls (even the sample with as low as 10% tumor cell content), it would be optimal to confirm their negative mutation status (wild-type) with another sensitive molecular method (e.g., pyrosequencing or droplet digital (dd) PCR) for further validation of the Idylla system. Simultaneously, it should be emphasized that the Idylla BRAF mutation assay has previously been shown to be able to accurately detect *BRAF* mutational status in FFPE samples with even 2% tumor cell content [63].

In summary, we found a moderate BRAF V600E mutational frequency in our series of GGs and PAs, using a combination of BRAF-VE1 IHC on the Ventana stainer and real-time PCR-based BRAF mutation assay on the Idylla platform. We found this to be particularly accurate for pathology laboratories without molecular diagnostic units and in certain clinical situations demanding a rapid BRAF mutation analysis, given not only the similar merits of the assays but also the ability to complement each other's limitations. Both methods are CE-IVD labeled, easy to perform, fully automated, fast, cost-effective, and suitable for samples with a small quantity and low cellularity of tumor, as they have a low detection limit. BRAF-VE1 IHC appears even more suitable to characterize low*-*abundant tumor cells diluted in a large volume of the sample, but it detects only the V600E mutational variant, and the interpretation of staining results may be sometimes complicated and always requires the experienced pathologist. The Idylla BRAF mutation assay covers all the most prevalent and clinically relevant *BRAF* V600 mutations, and the interpretation of results is fully automatic. The present study showed a relatively good performance for BRAF-VE1 IHC in GG and PA with an occasional ambiguous or nonspecific staining in few samples, for which the Idylla molecular testing system allowed to determine the final results. Therefore, they seem to be complementary techniques also in the context of tissue-specific factors unique to brain tissue. However, their utility for neuropathology is highly dependent on validation of the Idylla technology on a larger cohort of CNS tumor patients against a reference method.

## Figures and Tables

**Table 1:** *BRAF* mutations detected by the Idylla BRAF mutation test.

Exon	Codon	Mutation	Nucleotide change	Protein	Genetic call
15	600	V600E	p.(Val600Glu)	(c.1799T>A)	V600E/E2/D
		V600E2	p.(Val600Glu)	(c.1799_1800delinsAA)	
		V600D	p.(Val600Asp)	(c.1799_1800delinsAT; c.1799_1800delinsAC)	
	600	V600K	p.(Val600Lys)	(c.1798 _ 1799delinsAA)	V600K/R/M
		V600R	p.(Val600Arg)	(c.1798 _ 1799delinsAG)	
		V600M	p.(Val600Met)	(c.1798G>A)	

**Table 2:** BRAF V600 mutations detected or not by Idylla™ in BRAF-VE1-positive or equivocal cases.

Sample no.	Histopathological diagnosis	Sex	Age	Tumor location	IHC staining intensity	Category of % stained cells	IRS	BRAF V600E Idylla
1	Ganglioglioma (WHO grade I)	M	23	Hippocampus	2+	4	8	V600E/V600E2/V600D
2	Ganglioglioma (WHO grade I)	M	51	Left temporal lobe	Equivocal			V600E/V600E2/V600D
3	Ganglioglioma (WHO grade I)	M	47	Left frontal lobe	Equivocal			WT
4	Pilocytic astrocytoma (WHO grade I)	F	31	Left hemisphere	1+	4	4	V600E/V600E2/V600D
5	Pilocytic astrocytoma (WHO grade I)	M	18	Left temporal lobe	2+	4	8	V600E/V600E2/V600D
6	Pilocytic astrocytoma (WHO grade I)	M	18	Left temporal lobe	2+	3	6	V600E/V600E2/V600D
7	Pilocytic astrocytoma (WHO grade I)	F	21	Cerebellar vermis	3+	3	9	V600E/V600E2/V600D
8	Pilocytic astrocytoma (WHO grade I)	M	22	Left temporal lobe	2+	4	8	V600E/V600E2/V600D
9	Pilocytic astrocytoma (WHO grade I)	M	31	Right temporal lobe	Equivocal			WT
10	Pilocytic astrocytoma (WHO grade I)	M	31	Lateral ventricle	Equivocal			WT

## Data Availability

The datasets used and/or analyzed during the current study are available from the corresponding author on reasonable request.
